# Changes in peripheral blood lymphocyte subsets during arthritis development in arthralgia patients

**DOI:** 10.1186/s13075-016-1102-2

**Published:** 2016-09-14

**Authors:** Joyce Lübbers, Marian H. van Beers-Tas, Saskia Vosslamber, Samina A. Turk, Sander de Ridder, Elise Mantel, John G. Wesseling, Martine Reijm, Ingrid M. van Hoogstraten, Johannes W. Bijlsma, Dirkjan van Schaardenburg, Hetty J. Bontkes, Cornelis L. Verweij

**Affiliations:** 1 Department of Pathology, VU University Medical Center, Amsterdam, The Netherlands; 2 Amsterdam Rheumatology and Immunology Center, Jan van Breemen Research Institute | Reade, Amsterdam, The Netherlands; 3 Amsterdam Rheumatology and Immunology Center, VU University Medical Center, Amsterdam, The Netherlands; 4 Amsterdam Rheumatology and Immunology Center, Academic Medical Centre, Amsterdam, The Netherlands; 5 Department of Oral Cell Biology, ACTA, Amsterdam, The Netherlands; 6 Department of Pathology, VU University Medical Center, Inflammatory Disease profiling Unit, CCA2.21, P.O. box 7075, Amsterdam, 1007MB The Netherlands

**Keywords:** Rheumatoid arthritis, Arthralgia, Cell subsets, Flow cytometry

## Abstract

**Background:**

Multiple lymphocyte subsets like T and B cells have been connected to joint infiltration and inflammation in rheumatoid arthritis (RA). Identification of leucocyte subsets that are dysregulated in arthritis development could provide insight into the aetiology of RA. This study aimed to investigate the composition of the peripheral blood components, i.e. CD14^+^ monocytes, CD4^+^ and CD8^+^ T lymphocytes (CD3^+^), CD80^+^, C-X-C chemokine receptor 3 (CXCR3)^+^ and CD27^+^ B lymphocytes (CD19^+^), CD16^+^CD56^+^CD3^−^ natural killer (NK) cells and activated CD56^+^CD3^+^ T cells, for association with arthritis development in patients with arthralgia.

**Methods:**

Peripheral blood was collected from 89 patients with early RA (disease duration <6 months), 37 healthy controls (HC) and 113 patients with arthralgia (22 developed arthritis within ≤1 year, 18 developed arthritis after >1 year and 73 did not develop arthritis). Absolute numbers of monocytes and lymphocyte subsets in whole heparinized blood were determined with flow cytometry using quantification beads in combination with fluorescent labelled antibodies for T cells, B cells, monocytes, NK cells and activated T cells.

**Results:**

In patients with early RA, significant decreases in numbers of (activated) T cells, CD80^+^ and memory B cells and a trend towards smaller numbers of CD8^+^ T cells was observed compared to HC. Similar differences were seen in patients with arthralgia who developed or did not develop arthritis (non-converters), with significantly decreased CD8^+^ T cells and memory B cells. Patients with arthralgia who developed arthritis were split into groups that developed arthritis within 1 year (early converters) or after 1 year (late converters). Late converters had a significantly decreased number of CD8^+^ T cells compared to non-converters; early converters had a decreased number of memory B cells. Longitudinal analysis of converters showed a significant relative increase in CD80^+^ B cells towards the conversion time point compared to 24 months prior to conversion.

**Conclusions:**

This study revealed that patients with arthralgia who develop arthritis demonstrate a change in cellular immune parameters apparent in the periphery, starting with a decrease in cytotoxic T cells 24 months prior to arthritis development, followed by a decrease in the number of memory B cells 12 months prior to disease onset.

**Electronic supplementary material:**

The online version of this article (doi:10.1186/s13075-016-1102-2) contains supplementary material, which is available to authorized users.

## Background

Rheumatoid arthritis (RA) is an autoimmune disease affecting the joints. Inflammation of the joints leads to destruction of bone and cartilage in the joint [1]. Although the aetiology of the disease is still unknown it is likely that inadvertent immune activation is the basis for the development of RA. Understanding the immune mechanism and components that lead to chronic joint inflammation is of utmost importance to allow timely identification and treatment in order to prevent arthritis development and concomitant joint destruction.

Among these immune components are autoantibodies against citrullinated peptides (APCA) and rheumatoid factor (RF), which are seen years before the clinical manifestation of RA [2, 3]. More recently we have identified the presence of the type I interferon (IFN) signature in peripheral blood cells in 52 % of patients with arthralgia who developed arthritis within 2 years [4]. Moreover, we provided evidence that a decreased B cell count, corresponding to lower memory B cell numbers, in the periphery, is associated with arthritis development [5].

In this study we analysed the peripheral blood components, i.e. CD14^+^ monocytes, CD4^+^, CD8^+^ and CD56^+^T lymphocytes (CD3^+^), CD80^+^, C-X-C chemokine receptor 3 (CXCR3)^+^, CD27^+^ B lymphocytes (CD19^+^) and CD16^+^CD56^+^CD3^−^ NK cells, for their association with arthritis development. We therefore, studied individuals who were seropositive and had joint complaints without swelling of the joints, i.e. patients defined as having arthralgia. These patients were followed until the development of arthritis, defined as having at least one swollen joint. Furthermore, we investigated the variability of the frequency of the different cell subsets over the course of arthritis development in these patients.

## Methods

### Study population

The Amsterdam Reade cohort used for this study consisted of 113 patients with arthralgia of whom 22 developed arthritis within 1 year (converters ≤12 months) and 18 developed arthritis after 1 year (converters >12 months). Inclusion criteria for the patients with arthralgia were positivity for ACPA and/or RF [6], joint complaints without clinical arthritis determined by two independent rheumatologists and a minimum follow-up period of 24 months. Exclusion criteria for the patients with arthralgia were the (previous) use of disease-modifying anti-rheumatic drugs (DMARDs), a history of arthritis and evidence of erosions on radiographs. Arthritis was defined as having one or more swollen joints as assessed by two independent rheumatologists. Furthermore, 37 healthy controls (HC) and 89 patients with early RA were included at Reade. Inclusion criteria for the patients with early RA were disease duration <6 months and no previous use of DMARDS or biological agents. See Table 1 for demographic and clinical characteristics of all study groups.

**Table 1:** Demographic characteristics of healthy controls, patients with arthralgia and patients with early RA

	Healthy controls (HC)	Patients with arthralgia				Patients with early RA
		All patients with arthralgia	Arthritis development <1 year (early converters)	Arthritis development >1 year (late converters)	Did not develop arthritis (non-converters)	
Individuals (*n*)	37	113	22	18	73	89
Median age at inclusion in years (IQR)	30 (27–41)	50 (41–57)	55 (32–65)	45 (39–57)	51 (41–58)	53 (42–60)
Female (*n*) (%)	16 (64)	86 (76)	17 (77)	14 (78)	55 (75)	59 (66)
ACPA positive (*n*) (%)	N.D.	69 (61)	18 (82)	16 (89)	35 (48)	53 (60)^a^
RF positive (*n*) (%)	N.D.	50 (44)	12 (55)	5 (28)	33 (45)	49 (55)^a^
ACPA and RF positive (*n*) (%)	N.D.	29 (26)	11 (50)	6 (33)	12 (16)	43 (48)^a^
Median follow-up time in months (IQR)	N.A.	26.9 (19.1–42.4)	8.1 (1.3–12.3)	22.3 (13.9-41.6)	36 (25.7–47.9)	N.A.
Median time to arthritis development in months (IQR)	N.A.	12.48 (7.4–22.1)	8.1 (1.3–12.3)	22.3 (13.9–41.6)	N.A.	N.A.

^a^Available for 87 out of 89 patients. *RA* rheumatoid arthritis, *HC* healthy controls, *N.A*. not applicable, *N.D*. not determined, *ACPA* anti-citrullinated peptide antibodies, *RF* rheumatoid factor, *IQR* interquartile range

### Flow cytometry analysis

Absolute numbers and percentage of monocytes and lymphocyte subsets were determined with flow cytometry (FACS Calibur) on whole heparinized blood. Quantification beads (Trucount, BD) in combination with CD45 fluorescein isothiocyanate (FITC), CD14 phycoerythrin (PE), CD3 peridinin chlorophyll protein (PerCP) and CD19 allophycocyanin (APC) were used to measure absolute numbers of lymphocytes, monocytes, B cells and T cells according to the manufacturer’s instructions. Absolute numbers of cell subsets were calculated using the percentage of cells within a main cell type that was measured with quantification beads. For the subsets CD45 and CD3 or CD19 were always taken in multiple tubes combined with the following markers: CD8 PE, CD4 APC, CD16/56 PE, CXCR3 APC, CD80 PE and CD27 FITC (all products from BD Biosciences, San Jose, CA, USA). Flow cytometry data were analysed using FACSDIVA software version 6.1.3. Forward, sideward scatter and CD45^bright^ were used to select lymphocytes. CD16^+^CD56^+^CD3^−^ cells were defined as natural killer (NK) cells and CD16/CD56^+^ CD3^+^cells are expected to be predominantly CD56^+^CD16^−^. As CD56^+^CD3^+^ T cells, also described as NK T-like cells, have been described as activated effector cells [7, 8] we define them here as activated T cells. Gating strategy is depicted in Additional file 1: Figure S1.

### Statistical analyses

GraphPad Prism 5.0 was used for statistical analysis. The Mann-Whitney *U* test or Kruskal-Wallis test followed by Dunn’s multiple comparison test were used to compare the different parameters between patient groups. Log^2^ values were used for the ratios, showing the timeframe from conversion time point and 12 or 24 months prior to conversion. The one sample *t* test or Wilcoxon signed rank test was used to test if the median was significantly different from 0, which would indicate an increase or decrease from the conversion time point. *P* values <0.05 were considered to be significant.

## Results

**Fig. 1:**
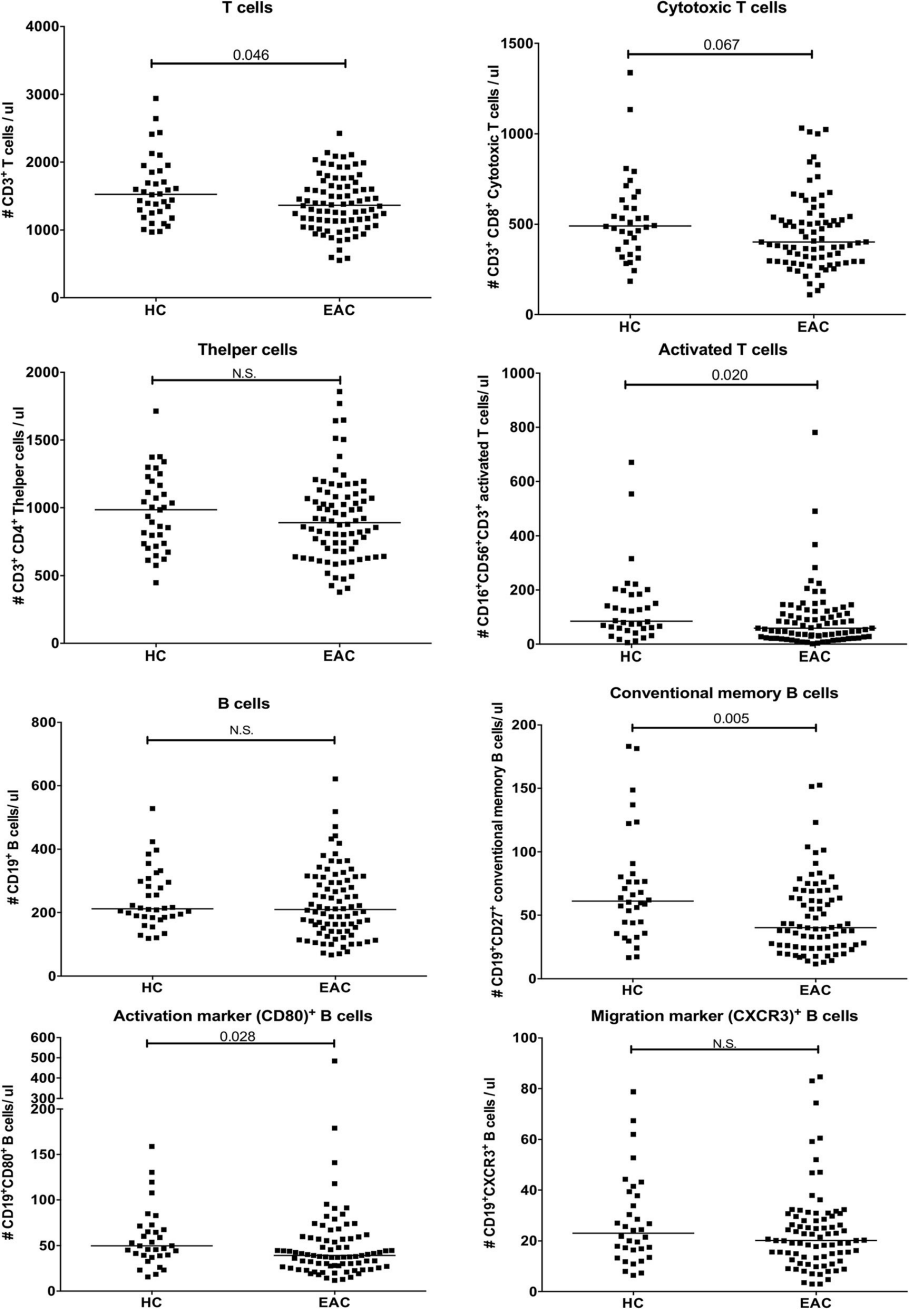
Absolute numbers of cells in the lymphocyte subsets in 89 patients with early rheumatoid arthritis (*EAC*) and 37 healthy controls (*HC*). *Black line* represents the median of the whole group. Differences between groups were tested using the Mann-Whitney *U* test. *N.S*. not significant

In order to gain insight into the immune differences between HC and patients with early RA, the number of circulating monocytes, lymphocytes, NK cells, activated T cells, B cells, T cells and B and T cell subsets were assessed by flow cytometry. This revealed that patients with early RA had a significantly lower overall number of circulating CD3^+^ T cells, CD3^+^CD56^+^CD16^+^ activated T cells, conventional memory (CD27^+^) B cells and activated (CD80^+^) B cells compared to HC (Fig. 1). There was a similar trend in the number of CD8^+^ cytotoxic T cells. The number of monocytes (data not shown), overall lymphocytes (data not shown), CD4^+^ T helper cells, B cells and migration marker (CXCR3)^+^ B cells did not differ between HC and patients with early RA. This indicates that memory B cells may have left the circulation in patients with early RA.

**Fig. 2:**
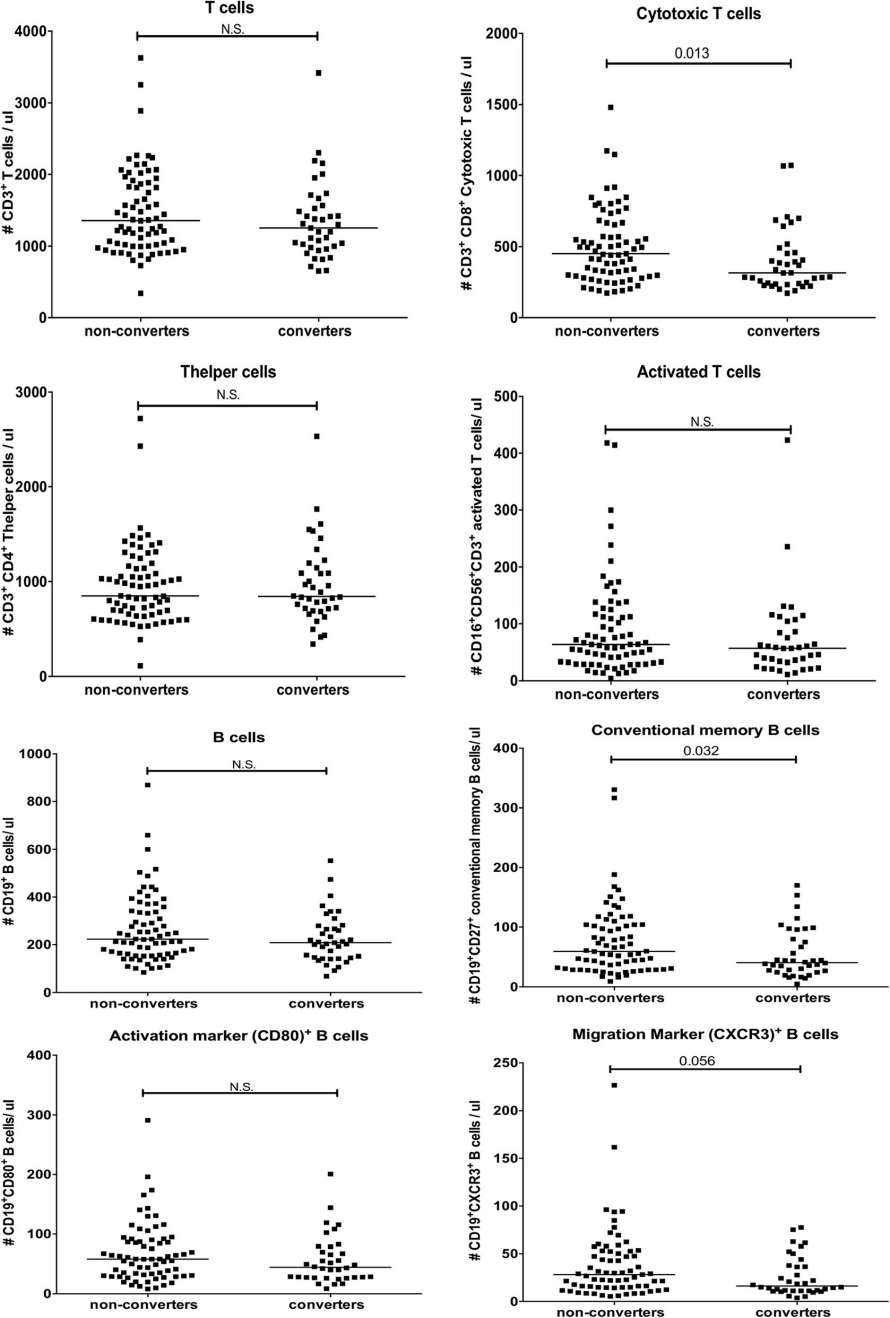
Absolute numbers of cells per lymphocyte subset in 40 patients with arthralgia who developed arthritis within 5 years (converters) and 73 patients with arthralgia who did not develop arthritis (non-converters). *Black line* represents median of the whole group. Differences between groups were tested using the Mann-Whitney *U* test. *N.S*. not significant

Next we compared the same lymphocyte subsets at the inclusion time point in patients with arthralgia who developed arthritis within 5 years (converters) with patients with arthralgia who did not develop arthritis (non-converters). This revealed that converters had a significant lower number of CD8^+^ cytotoxic T cells and CD27^+^ memory B cells compared to non-converters (Fig. 2). There was no significant decrease in migration marker (CXCR3)^+^ B cells between non-converters and patients with early arthritis (*p* = 0.18). A significant decrease was observed in CD27^+^ memory B cells in patients with early RA compared to non-converters and HC (*p* = 0.008 and *p* = 0.036, respectively). Differences between HC, non-converters, converters and patients with early arthritis are depicted in Additional file 2: Figure S2. This indicates that some of the difference in the immunological status of patients with early RA have already occurred in converters by the first visit to the rheumatologist.

**Fig. 3:**
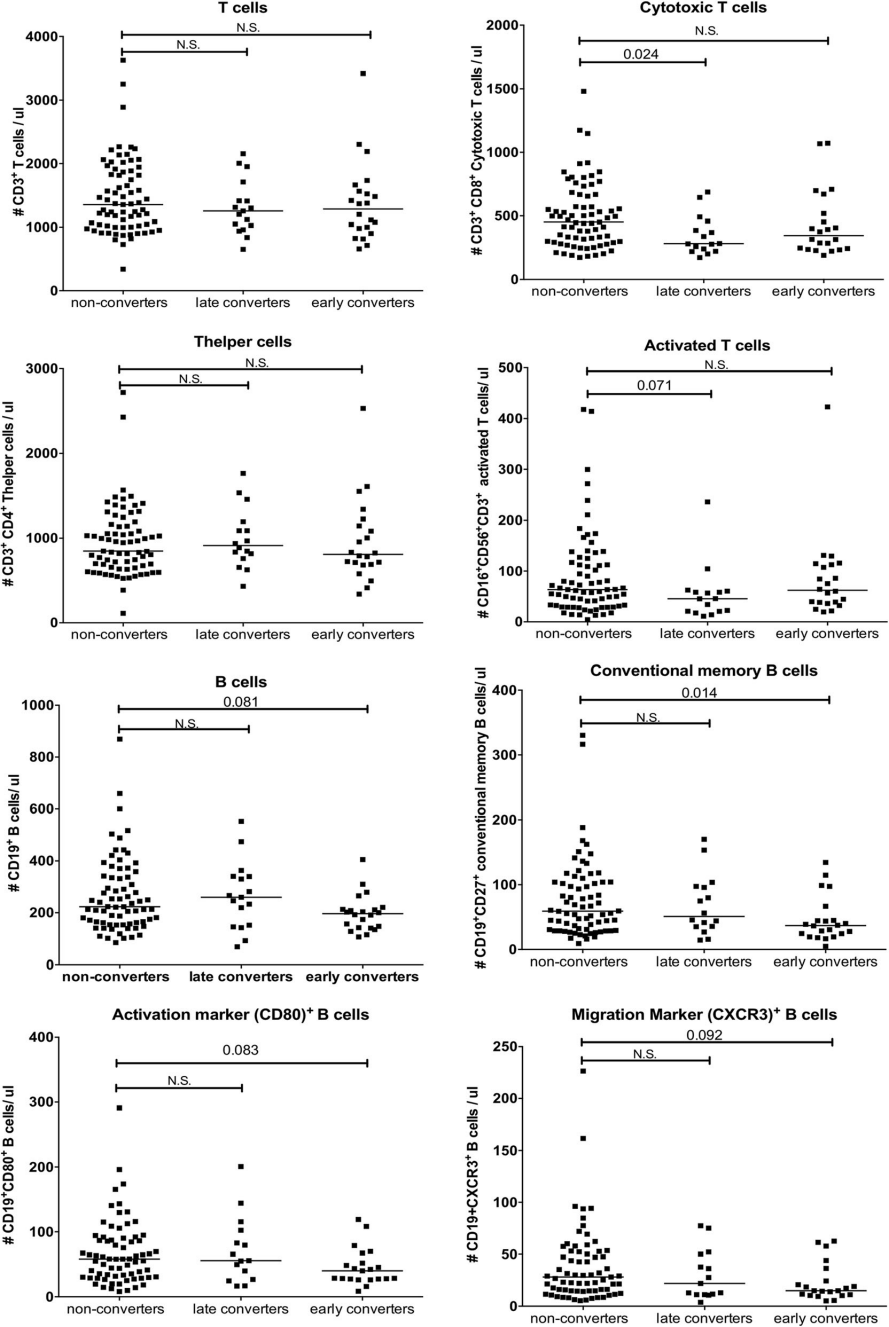
Absolute numbers of cells per lymphocyte subset in 73 patients with arthralgia who did not develop arthritis (non-converters), 22 who developed arthritis within a year (early converters) and 18 who developed arthritis after 1 to 5 years (late converters). *Black line* represents the median of the whole group. Differences between groups were tested using the Kruskal-Wallis test followed by Dunn’s multiple comparison test. *N.S*. not significant

In the Amsterdam Reade cohort, the patients with arthralgia were followed by a rheumatologist for research purposes, for a maximum of 5 years, to determine whether arthritis developed. Therefore, in this cohort, arthritis could occur between the time point of inclusion and at a maximum follow up of 5 years. To investigate whether there was a difference in the composition of circulating mononuclear cells at the inclusion time point between patients with arthralgia who developed arthritis within a year and between 1 and 5 years, the converters were divided into early and late converter groups. This revealed that the late converters had a significantly lower number of CD8^+^ cytotoxic T cells compared to non-converters (Fig. 3). No differences between late converters and non-converters were observed for B cells, memory B cells or activation and migration markers. In the early converters there was a significant decrease in the number of memory B cells (Fig. 3). The same trend was visible for the overall number of B cells and the activation and migration marker-positive B cells.

**Fig. 4:**
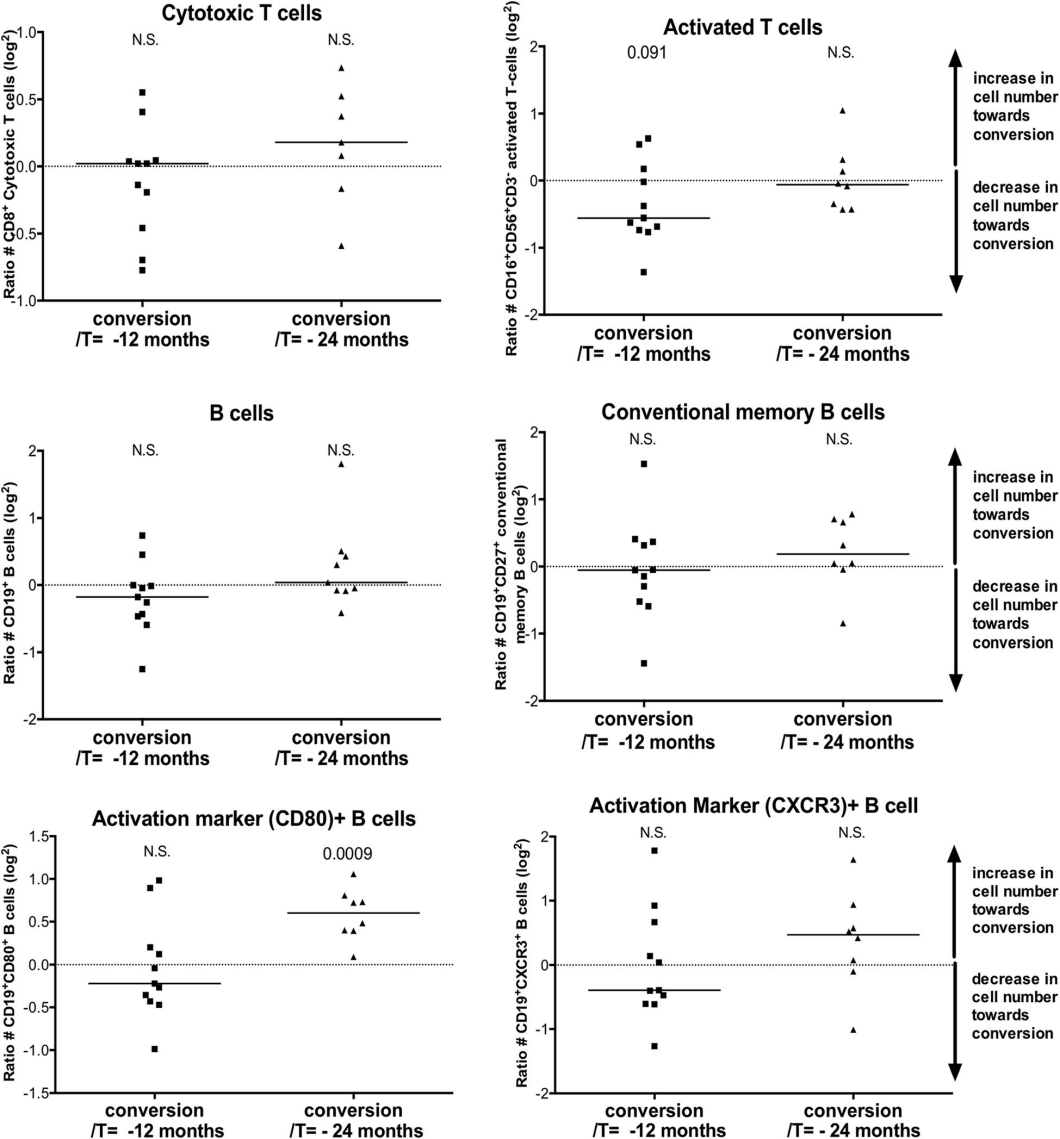
Negative log^2^ ratios of the absolute number of cells per lymphocyte subset at the time of arthritis development (conversion) divided by the absolute number of cells per lymphocyte subset 12 or 24 months prior to arthritis development in 17 patients with arthralgia who developed arthritis. A ratio *>0* indicates increased cell number at 12 or 24 months before arthritis development compared to the conversion time point. *Black line* represents the median of the whole group. Differences between the conversion time point and 12 or 24 months prior to arthritis development (e.g. difference from 0) was tested using the one-sample *t* test or Wilcoxon signed rank test. *P <0.05, **P <0.01 and ***P <0.001. *N.S*. not significant

The data as described suggest that cytotoxic T cells and memory B cells leave the periphery before the clinical diagnosis of arthritis. To gain more insight into the time frames of action for these cell types, we investigated a small group of 17 converters using flow cytometry measurements at the time point of conversion and 12 and/or 24 months prior to conversion. Additional file 3: Figure S3 depicts the changes over time in both converting and 68 non-converting patients with arthralgia. To more clearly depict the changes in converters at 12 or 24 months prior to conversion, log^2^ ratios were calculated to show the relative increase or decrease compared to the conversion time point. This revealed that there was a broad distribution within the cell types, where one patient showed a relative increase towards conversion and the other revealed a relative decrease towards the conversion time point (Fig. 4). The most outstanding cell types were the activation-marker-positive B cells, which showed a significant relative increase towards the conversion time point compared to 24 months prior to conversion. This may indicate that in the very early stages, possibly years before the development of arthritis, cytotoxic T cells and conventional memory B cells migrate towards the lymph nodes or the joints.

## Discussion

Changes in immune parameters and B cells in particular, have been extensively studied in the peripheral blood of patients with RA in relation to therapy response, pathology of RA and clinical parameters. In patients with early RA, a decrease in total memory B cells and an increase in serum markers for the activation of B cells such as beta2 microglobulin have been described [9, 10]. We have recently described lower numbers of memory B cells as a potential biomarker to predict arthritis development in patients with arthralgia [5]. In the present study, we demonstrate that changes in peripheral blood mononuclear subsets, especially a smaller number of CD8^+^ T cells were seen 24 months prior to developing arthritis, whereas 12 months prior to arthritis development, the number of memory B cells was lower compared to patients with arthralgia who did not develop arthritis.

Our data demonstrate that there is a clear difference between patients with early RA and HC, with a decrease in CD8^+^ cytotoxic T cells, CD3^+^CD56^+^CD16^+^ activated T cells, memory B cells and activated B cells in patients with early RA. This could be partly due to the age differences between the HC and the patients with early RA, as it patients with established RA have a prematurely aged immune system that is about 20 years older than the healthy controls [11]. However, our data suggest that these changes start early in the development of RA, as converters with arthralgia display similar changes. Late converters had smaller numbers of CD8^+^ cytotoxic T cells, whereas the early converters had a decrease in memory B cells. The smaller number of memory B cells found in the peripheral blood of early converters and patients with early RA is similar to what is described by McComish et al. for DMARD-naïve patients with early RA [12]. This reduced number could be due to migration of memory B cells to the synovium of the affected joints.

We observed trends towards decreased activated B cells and CXCR3^+^ B cells, which suggests increased migration of activated B cells to the joints. These populations and CD27^+^ memory B cells may be partly overlapping [13]. Increased migration of these subsets is in line with the study of Nanki et al. describing more memory B cells and CXCR3-positive B cells in the synovium compared to the peripheral blood in patients with early RA [14]. Alternatively, B cells may have migrated towards the lymph nodes or bone marrow. In patients with early RA significantly more B cells were observed in the draining lymph nodes of the inflamed joints than in similar lymph nodes among HC [15]. In patients with established RA the bone marrow adjacent to the affected joints reveals a cell-rich inflammatory environment instead of a fat-rich non-inflammatory environment as seen in HC [16]. Both the increase in cells in the lymph nodes of patients with arthralgia and a more inflammatory environmebn in the bone marrow adjacent to the affected joints in patients with RA point towards a systemic immune activation in patients with arthralgia in whome B cells may migrate towards the secondary lymphoid organs, bone marrow or joints approximately a year before clinical signs arise.

Reduced numbers of CD8^+^ cytotoxic T cells are associated with early arthritis, as there was a trend towards lower numbers in the patients with early arthritis and a significant reduction in (late) converters compared to non-converters. It has previously been described that in patients with established RA no significant differences were found in T cell subsets compared to HC;, however there was a shift seen towards more CD8+ terminally differentiated effector memory T cells [17]. This might point towards early activation of cytotoxic T cells in arthralgia and in patients with early RA, which may later differentiate into the terminally differentiated effector memory T cells found in patients with established RA. There are indications that there is increased migration of CD8^+^ cytotoxic T cells to the lymph nodes and joints in patients with RA. In the lymph nodes of patients with early RA and patients with arthralgia who did not develop RA, there were no differences in the frequency of total CD8^+^ cytotoxic T cells. However, there was a significant increase in activated CD69^+^ CD8^+^ T cells in the lymph nodes of patients with early R, and a tendency towards this in patients with arthralgia [15]. de Hair et al., described subtle infiltration of CD8^+^ cytotoxic T cells in the synovium of patients with arthralgia [18]. These data suggest that cytotoxic T cells in patients with arthralgia become more activated in the lymph nodes before migrating towards the affected joints, which might accelerate the inflammation in the joint by producing more pro-inflammatory cytokines [19]. No differences were observed in CD4^+^ T helper cells between HC, non-converters, converters and patients with early RA. This is in concordance with a recent publication on CD4^+^ T cells, reporting no differences in the absolute number of CD4^+^ T cells or in CD4^+^ (terminally differentiated) effector memory cells between HC and seropositive patients with arthralgia. An increase in CD4^+^CD161^+^ T cells expressing a TH17 or TH1/TH17 phenotype was observed in seropositive patients with arthralgia compared to HC, suggesting a role for TH17 cells in RA development [20]. Our data suggest that increased migration of B cell subsets and CD8^+^ cytotoxic T cells is reflected by decreased numbers of these subsets in peripheral blood; however, larger studies are necessary to confirm this.

Recently seropositive patients with arthralgia were reported to have a decreased percentage of NK cells, especially CD56^dim^ NK cells, compared to HC [21]; however, there were no differences in absolute numbers of NK cells within the CD45^+^ pool. In our comparison of converting and non-converting seropositive patients with arthralgia there was no decrease in the absolute numbers or percentage (data not shown) of overall NK cells; however, there was no sub-classification of NK cells on CD56^dim^ or CD56^bright^. Furthermore, no differences in NK cells between HC, non-converting and converting patients with arthralgia and patients with early RA were observed. This could be due to differences in patient population and inclusion criteria for the patients with arthralgia.

Longitudinal data for the patients with arthralgia among both non-converters and converters show that there was fluctuation in the percentage of all immune cells that we measured. This could be a reflection of the normal fluctuation that we also observed in HC or due to seasonal infections like the influenza virus; however, patients with symptoms of influenza were asked to return at a later time point for blood collection. What emerged from the longitudinal data, although from a small cohort, is the significant increase in the number of activated B cells within patients between 12 and 24 months before conversion. Our study provides indications of which immune cell subsets and changes therein are involved in the phase preceding development of clinical RA. Larger studies are needed to better understand the time points of these changes, as well as the transitions between the blood, bone marrow, lymph node and synovial compartments. These studies will enable more precise prediction for clinical use and the definition of time points and targets for specific interventions.

## Conclusions

The main conclusion from this study is that patients with arthralgia who develop arthritis have changes in circulating lymphocyte subsets, starting with a decrease in CD8^+^ cytotoxic T cells 24 months prior to arthritis development, followed by a decrease in the number of activated memory B cells 12 months prior to disease onset.

## Additional files

Additional file 1:
**Figure S1.** Gating strategy of the flow cytometry analysis. **A** Gating strategy with quantification beads. Gate 1 for living cells, which are depicted in the second dot plot. Gate 2 for lymphocytes, gate 3 for monocytes and gate 4 for the quantification beads. CD3 and CD19 positivity was determined in the lymphocyte gate and depicted in *plot 5*. **B** Gating strategy for the immunological subsets. Lymphocyte selection based on FSC and SSC properties (*plot 1*) followed by selection on CD45 properties (*plot2*). All the subsets were determined in the lymphocyte gate. (TIF 3403 kb) Additional file 2:
**Figure S2.** Dot plots of the absolute number of cells in the lymphocyte subsets in 37 healthy controls (*HC*), 40 patients with arthralgia who developed arthritis within 5 years (converters), 73 patients with arthralgia who did not develop arthritis (non-converters) and 89 patients with early RA (*EA*C). The *black line* represents median of the whole group. Differences between groups were tested with the Kruskal-Wallis test followed by Dunn’s multiple comparison test. *N.S*. not significant. (TIF 791 kb) Additional file 3:
**Figure S3.** Line plot of the absolute number of cells per lymphocyte subset in 68 non-converting and 17 converting patients with arthralgia. The non-converting patients are depicted from the time point of inclusion. The converting patients are depicted from the time point of conversion and the time before conversion. (TIF 1784 kb)

## Abbreviations

ACPA: anti-citrullinated peptide antibodies
APC: allophycocyanin
CXCR3: C-X-C chemokine receptor 3
DMARDs: disease-modifying anti-rheumatic drugs
FITC: fluorescein isothiocyanate
HC: healthy controls
IFN: interferon
MFI: mean fluorescent intensity
NK: natural killer
PE: phycoerythrin
PerCP: peridinin chlorophyll protein
RA: rheumatoid arthritis
RF: rheumatoid factor
