# Supplementary figures and images for: Changes in peripheral blood lymphocyte subsets during arthritis development in arthralgia patients
Source: Arthritis Res Ther. 2016 Sep 14;18:205. doi: 10.1186/s13075-016-1102-2 (PMC5024500; doi:10.1186/s13075-016-1102-2)

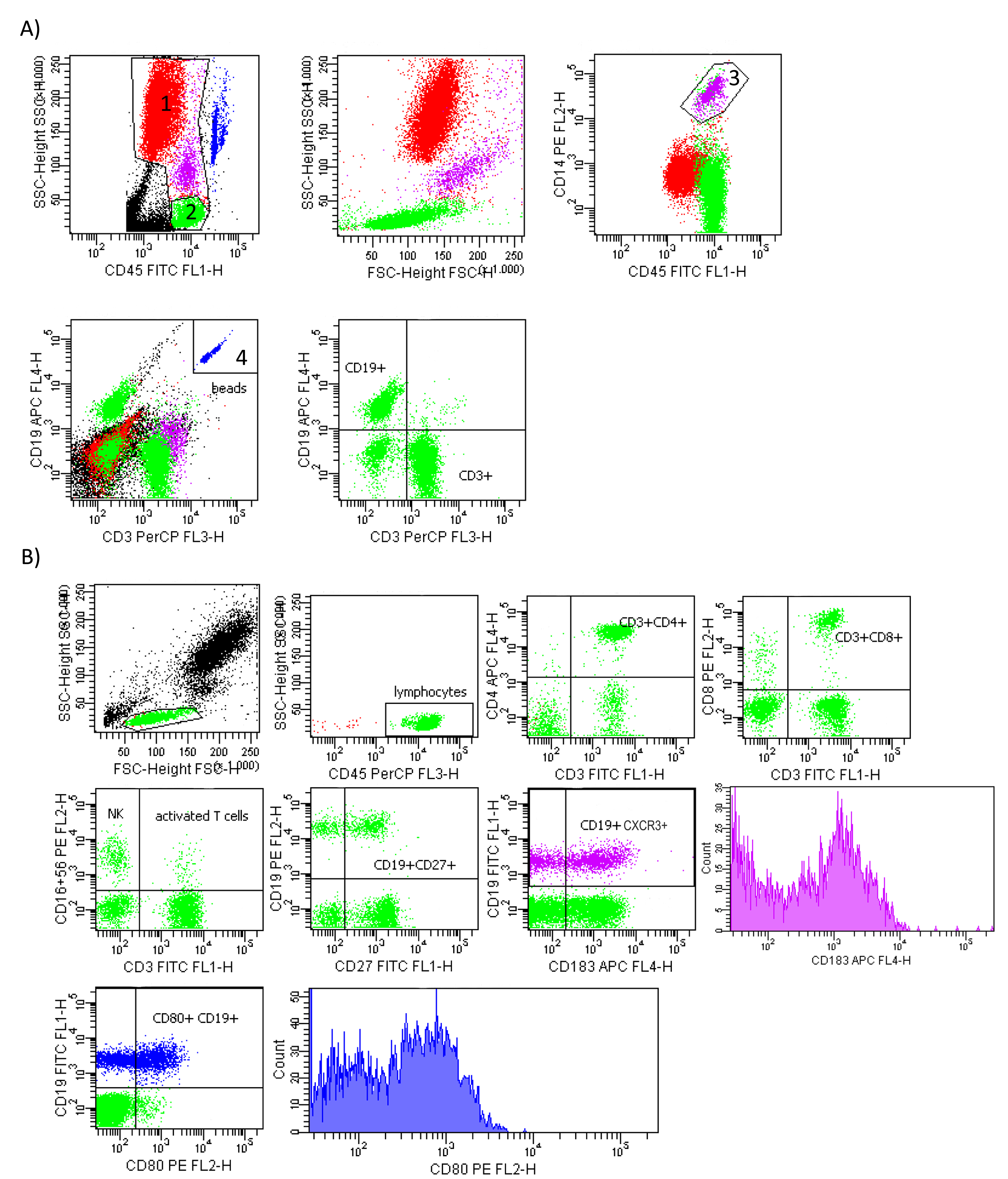

Supplement: Additional file 1: — Figure S1. Gating strategy of the flow cytometry analysis. A Gating strategy with quantification beads. Gate 1 for living cells, which are depicted in the second dot plot. Gate 2 for lymphocytes, gate 3 for monocytes and gate 4 for the quantification beads. CD3 and CD19 positivity was determined in the lymphocyte gate and depicted in plot 5. B Gating strategy for the immunological subsets. Lymphocyte selection based on FSC and SSC properties (plot 1) followed by selection on CD45 properties (plot2). All the subsets were determined in the lymphocyte gate. (TIF 3403 kb) [file 13075_2016_1102_MOESM1_ESM.tif]

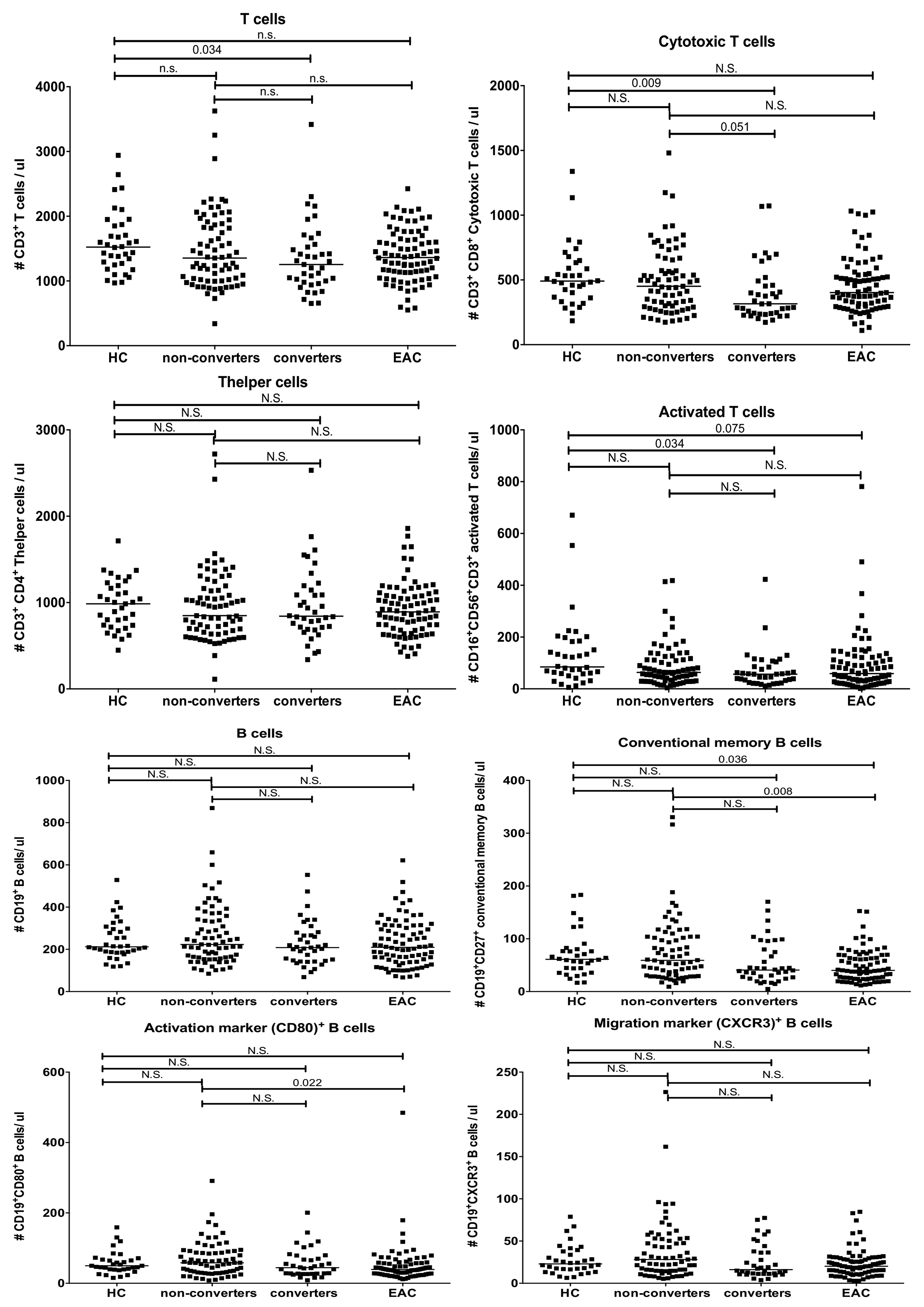

Supplement: Additional file 2: — Figure S2. Dot plots of the absolute number of cells in the lymphocyte subsets in 37 healthy controls (HC), 40 patients with arthralgia who developed arthritis within 5 years (converters), 73 patients with arthralgia who did not develop arthritis (non-converters) and 89 patients with early RA (EAC). The black line represents median of the whole group. Differences between groups were tested with the Kruskal-Wallis test followed by Dunn’s multiple comparison test. N.S. not significant. (TIF 791 kb) [file 13075_2016_1102_MOESM2_ESM.tif]

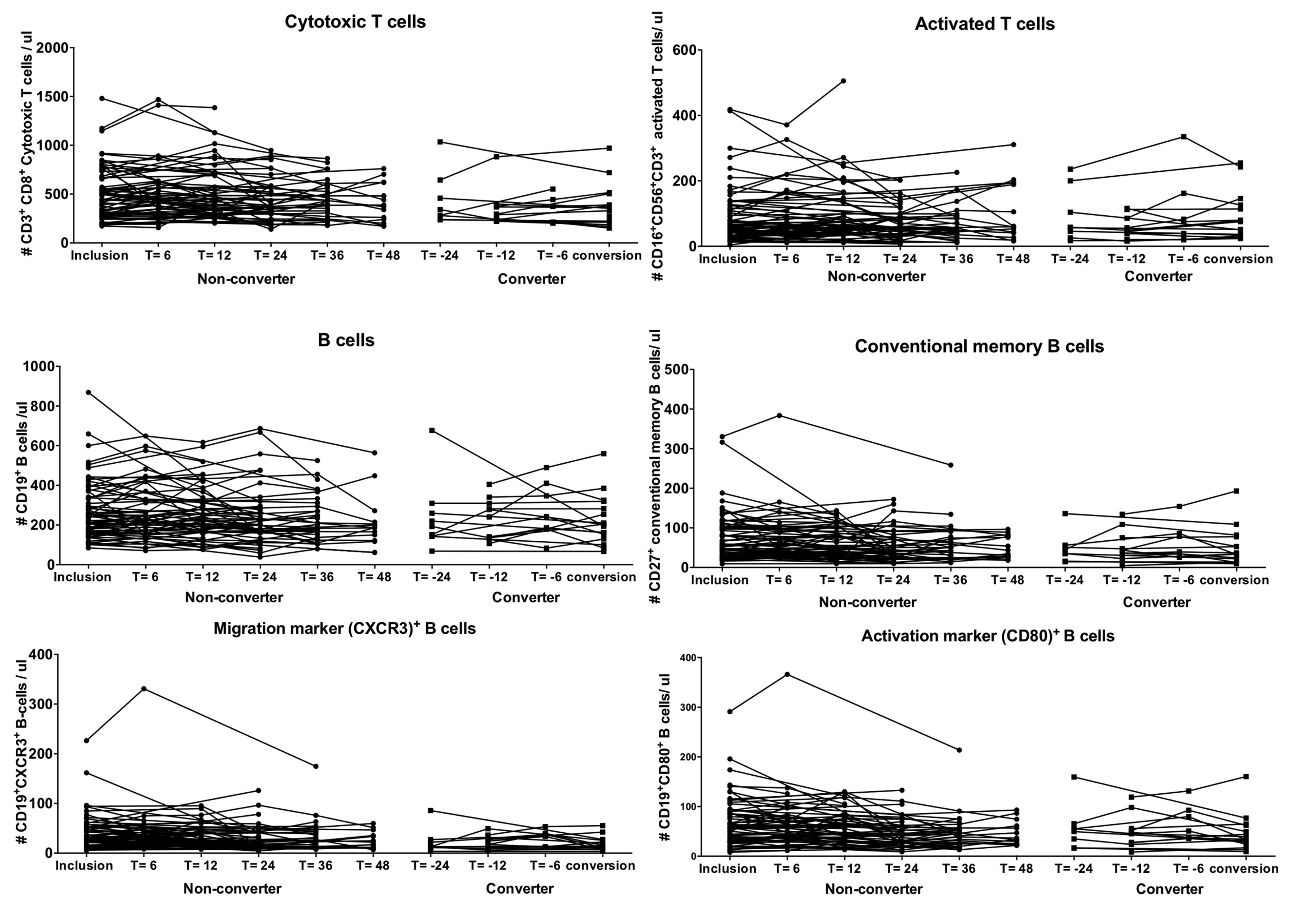

Supplement: Additional file 3: — Figure S3. Line plot of the absolute number of cells per lymphocyte subset in 68 non-converting and 17 converting patients with arthralgia. The non-converting patients are depicted from the time point of inclusion. The converting patients are depicted from the time point of conversion and the time before conversion. (TIF 1784 kb) [file 13075_2016_1102_MOESM3_ESM.tif]
